# Evaluation of safety and effectiveness of remdesivir in treating COVID-19 patients after emergency use authorization study

**DOI:** 10.3389/fphar.2023.1205238

**Published:** 2023-06-30

**Authors:** Erlina Burhan, Elisna Syahruddin, Fathiyah Isbaniah, Ginanjar Arum Desianti, Fanny Fachrucha, Cut Yulia Indah Sari, Efriadi Ismail, Puji Astuti, Muhammad Farhan Maruli, Farhan Mubarak, Anggit Tresna Rengganis, Hazia Hanifa Bilqis, Imammurahman Taslim, Evan Sastria, Elvan Wiyarta

**Affiliations:** ^1^ Department of Pulmonology and Respiratory Medicine, Persahabatan Central General Hospital, Universitas Indonesia, Jakarta, Indonesia; ^2^ Department of Pulmonology, Jakarta Islam Hospital Cempaka Putih, Jakarta, Indonesia; ^3^ Department of Pulmonology, Yarsi Hospital, Jakarta, Indonesia; ^4^ Department of Pulmonology, Cengkareng District General Hospital, Jakarta, Indonesia; ^5^ General Practitioner, Yarsi Hospital, Jakarta, Indonesia; ^6^ Department of Medical Science, Faculty of Medicine, Universitas Indonesia, Jakarta, Indonesia

**Keywords:** coronavirus, mortality, ventilator, adverse effect, treatment outcome, length of stay, Indonesia

## Abstract

**Background:** This study aimed to determine the real-world safety and effectiveness of remdesivir in hospitalized adult COVID-19 patients with moderate-to-critical disease in Indonesia.

**Methods:** A multicenter, retrospective cohort study was conducted at four COVID-19 referral hospitals in Jakarta. A total of 587 patients were included, of whom 243 received remdesivir within 72 h of admission. The safety endpoints were the proportions of patients with any adverse event (AE), any grade 3 AE, and AE of each system organ class. The effectiveness endpoints were ICU admission >24 h from baseline, live discharge and mortality at day 14, live discharge and mortality at day 28, and virologic conversion. Patients who received remdesivir within 72 h of admission were considered the treatment group, and those who did not were the control group. Multivariate adjustments were performed using a modified Poisson regression.

**Results:** The study found no significant differences in safety endpoints between the two groups. However, the effectiveness endpoints showed that remdesivir was associated with a decreased risk of ICU admission >24 h from baseline (RR 0.71, 95% CI 0.52–0.96), an increased probability of live discharge at day 14 (RR 1.37, 95% CI 1.08–1.74), and an increased probability of live discharge at day 28 (RR 1.28, 95% CI 1.05–1.57). The rate of virologic conversion was not significantly different between the two groups.

**Conclusion:** The study concludes that remdesivir is safe and effective in the treatment of moderate-to-critical COVID-19 in a real-world setting in Indonesia.

## Introduction

Since the start of the Coronavirus Disease 2019 (COVID-19) pandemic, new and existing drugs have been proposed and studied one after another as potential therapeutics (Pan et al., 2021). One of these drugs is the nucleoside analogue prodrug remdesivir (GS-5734) (Sheahan et al., 2017). Previous *in vitro* and *in vivo* studies have shown that remdesivir inhibits the replication of various RNA viruses, including Ebola virus (EBOV), severe acute respiratory syndrome coronavirus (SARS-CoV), and Middle Eastern respiratory syndrome coronavirus (MERS-CoV) (Warren et al., 2016; Sheahan et al., 2017; Agostini et al., 2018). Soon after the virus’ emergence, *in vivo* inhibition of SARS-CoV-2 by remdesivir was demonstrated (Wang et al., 2020). Evidence of clinical efficacy, however, was unclear, notably with the Solidarity Trial finding little to no effect of remdesivir on hospitalized COVID-19 patients (Pan et al., 2021).

Remdesivir was first included in the Indonesian national treatment guideline for moderate-to-critical COVID-19 in August 2022 (Burhan et al., 2020), where previously only oseltamivir and favipiravir were recommended. The Indonesian Food and Drug Authority subsequently released an emergency use authorization (EUA) for remdesivir for COVID-19 in September 2022 (Food and Drug Authority of the Republic of Indonesia, 2020). With limited access to alternative therapeutics, remdesivir remains the preferred treatment for moderate-to-critical COVID-19 in Indonesia. Safety and efficacy data for remdesivir after EUA in Indonesia, however, are lacking. This study aimed to determine the real-world safety and effectiveness of remdesivir as a treatment for moderate-to-critical COVID-19.

## Methods

### Study design and setting

This is a multicenter, retrospective cohort study conducted at four COVID-19 referral hospitals in Jakarta (Persahabatan Central General Hospital, Jakarta Islamic Hospital, Yarsi Hospital, and Cengkareng District Hospital). We sought to evaluate the safety and effectiveness of remdesivir compared to usual treatment among adult hospitalized COVID-19 patients. All patients aged 18 years with confirmed COVID-19 (by polymerase chain reaction of a nasopharyngeal swab specimen) and moderate to critical disease as defined by the Indonesian National COVID-19 Management Guideline (Burhan et al., 2020) were consecutively included. Patients who received remdesivir prior to admission to the cohort hospital, received experimental treatment for COVID-19, died within 24 h of treatment, were referred to another hospital, or had incomplete medical records were excluded from this study. Although this might induce selection bias, we mitigate this with comprehensive statistical analysis. Ethical approval was sought from and obtained from the Ethics Committee of Persahabatan Hospital (64/KEPK-RSUPP/06/2021). Because of the retrospective design, the informed consent requirement was waived, and the data used in this study were anonymized before use according to the Declaration of Helsinski (World Medical Association, 2013).

Patients diagnosed with COVID-19 and treated with Remdesivir under an Emergency Use Authorization were included in this study. Remdesivir was administered in accordance with established protocols, beginning with a loading dose of 200 mg on the first day, followed by a maintenance dose of 100 mg beginning on the second day. The duration of treatment varied according to each patient’s clinical condition, but never exceeded 10 days. The clinical response of patients to the drug was monitored daily, including vital signs, laboratory values, and disease progression. Additionally, side effects and adverse reactions were meticulously recorded. In addition to receiving Remdesivir, patients received supplemental oxygen, antipyretics, and other treatments as determined by the discretion of the attending physicians. In order to control for potential confounding effects, we accounted for the use of other antiviral or immunomodulatory medications in our analysis.

Safety endpoints were proportions of patients with any adverse event (AE), any grade 3 AE, and AE of each system organ class (cardiovascular, respiratory, gastrointestinal, dermatologic, neurological, psychiatric, hepatobiliary, renal, hematologic, and metabolic). Effectiveness endpoints were intensive care unit (ICU) admission >24 h from baseline, live discharge and mortality at day 14, live discharge and mortality at day 28, and virologic conversion. To control for indication bias (e.g., patients receiving remdesivir only after clinical deterioration), patients who received remdesivir within 72 h of admission were considered the treatment group, and those who did not the control group.

### Data collection

Patient age, sex, medical history, clinical characteristics, oxygen supplementation requirement, medications, adverse events, and outcome were obtained from medical records. Laboratory data, which include serum alanine transaminase (ALT), aspartate transaminase (AST), blood urea nitrogen (BUN), serum creatinine, and estimated glomerular filtration rate (eGFR), were obtained from medical records and the hospital’s electronic laboratory information system. AE was defined as any untoward medical occurrence that is temporally associated with the studied treatment but with which it does not necessarily have a causal relationship. AEs were graded based on the Common Terminology Criteria for Adverse Events (CTCAE) Version 5.0 (Freites-Martinez et al., 2021).

### Statistical analyses

For bivariate analyses, categorical variables were compared by the Chi-squared test or Fisher’s exact test as appropriate; quantitative variables were compared by the Mann-Whitney-Wilcoxon test as appropriate. Categorical variables are presented as frequency and percentage, while numerical variables are presented as median and interquartile range (IQR). Effect sizes were given in risk ratios (RR) and 95% confidence intervals (CI). Multivariate adjustments were performed using a modified Poisson regression (Zou, 2004). All statistical analyses were performed using R version 4.1.3.

## Results

**FIGURE 1:**
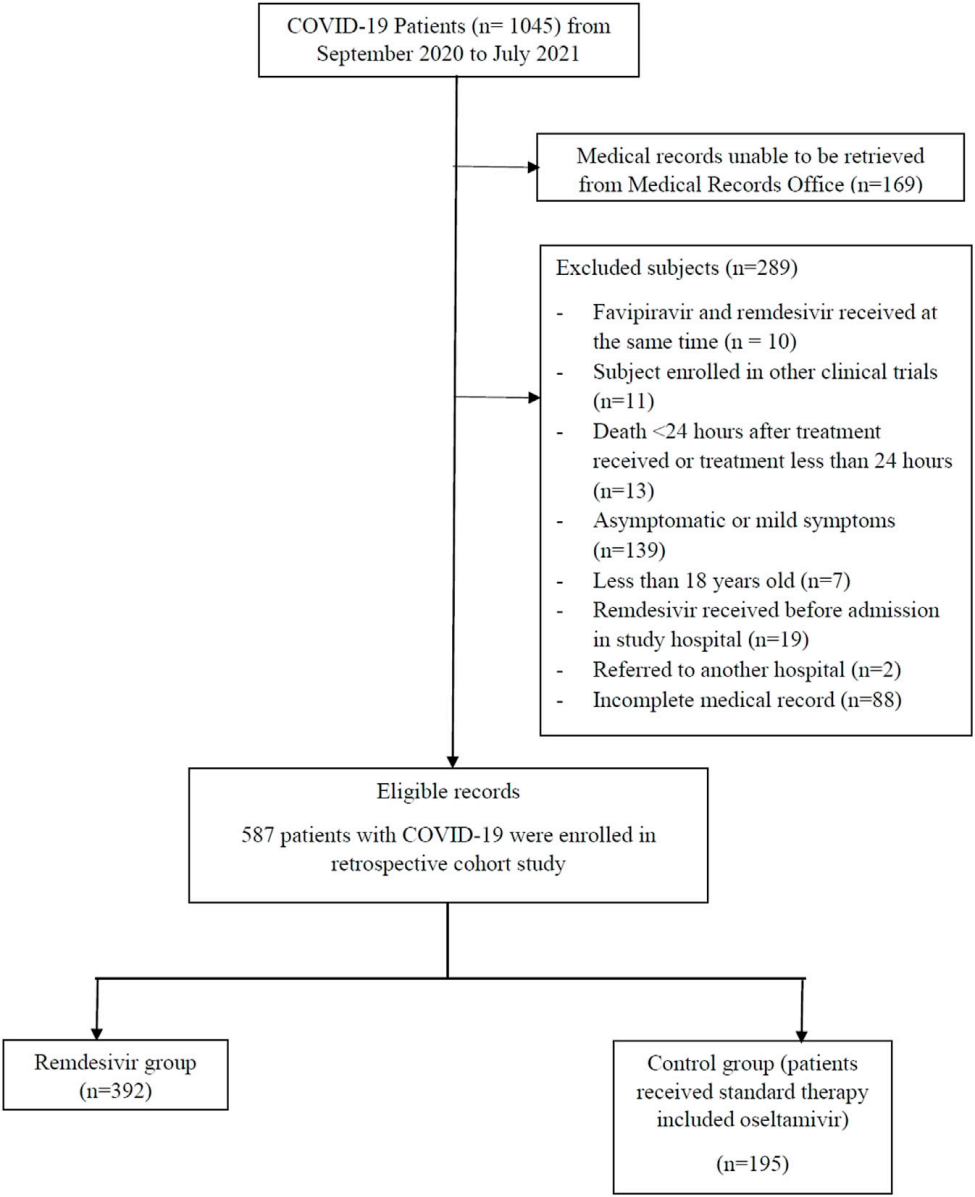
Eligibility assessment result.

A total of 1045 COVID-19 patients were admitted at the four sites between September 2020 and July 2021, of whom 899 met the inclusion criteria and 587 were eligible for analysis (Figure 1). Of these, 243 (41.4%) received remdesivir within 72 h of admission. The remaining 344 (58.6%) patients were started on oseltamivir. Those receiving remdesivir in the first 72 h were older, with a median age of 55 years (47–65), compared to the control group with a median age of 52 years (42–62). Those receiving remdesivir in the first 72 h were also more likely to be male (65.0% vs 55.5%) and have a more severe disease (54.7% vs 40.1%). Patients who received early remdesivir treatment were more likely to be obese (56.4% vs 47.1%), to have DM (48.1% vs 37.2%), and to have at least one cardiac or vascular disease (57.6% vs 49.1%) compared to the control group, as seen in Table 1.

**TABLE 1:** Baseline characteristics of the study participants.

Characteristics	N	Remdesivir	Oseltamivir	*p*-value
Clinical				
**Age, median (IQR)**	587	55 (46.0–65.0)	48 (36.0–60.0)	**<0.001^a^**
**Age, n (%)**				**0.007^a^**
**>60 y.o**	207	153 (39.0)	54 (27.7)	
**<60 y.o**	380	239 (61.0)	141 (72.3)	
**Sex, n (%)**				
**Male**	348	245 (62.5)	103 (53.1)	**0.029^a^**
**Female**	239	147 (37.5)	92 (46.9)	
**COVID-19 severity, n (%)**				
**Severe-critical**	271	221 (56.4)	50 (25.6)	**<0.001^a^**
**Mild-moderate**	316	171 (43.6)	145 (74.4)	
**BMI, median (IQR)**	587	25.7 (23.4–28.9)	24.2 (22.2–27.9)	**0.003^a^**
**BMI, n (%)**				**0.001^a^**
**≥25 kg/m2**	279	204 (53.0)	75 (38.9)	
**<25 kg/m2**	299	181 (47.0)	118 (61.1)	
**Physical**				
**Blood pressure, n (%)**				0.603
**≥140/90 mmHg**	192	131 (33.4)	61 (31.3)	
**<140/90 mmHg**	395	261 (66.6)	134 (68.7)	
**MAP, n (%)**				1.000
**<65 mmHg**	2	2 (0.5)	0 (0.0)	
**>65 mmHg**	585	398 (95.5)	187 (100.0)	
**Pulse, n (%)**				**0.008^a^**
**≥100 x/min**	158	119 (30.4)	39 (20.0)	
**<100 x/min**	429	273 (69.6)	156 (80.0)	
**Respiratory rate, median (IQR)**	587	24 (20.0–26.0)	21 (20.0–24.0)	**<0.001^a^**
**Laboratory**				
**ALT, median (IQR)**	517	44.0 (31.0–68.0)	31.0 (21.0–57.8)	**<0.001^a^**
**AST, median (IQR)**	517	42.0 (27.0–67.5)	32.0 (17.0–61.8)	**<0.001^a^**
**ALT and AST level, n (%)**				**<0.001^a^**
**Elevated**	365	257 (70.4)	70 (46.1)	
**Normal**	190	108 (29.6)	82 (53.9)	
**BUN, median (IQR)**	494	14.9 (10.8–21.5)	11.2 (7.5–19.5)	**<0.001^a^**
**Creatine, median (IQR)**	535	0.90 (0.70–1.20)	0.90 (0.70–1.18)	0.313
**eGFR, median (IQR)**	535	83.5 (60.8–106.0)	88.0 (64.4–112.0)	0.165
**cQT, median (IQR)**	354	413.0 (381.0–446.3)	422.5 (385.0–452.0)	0.183
**Medication (yes/no)**				
**Azithromycin, n (%)**	460	318 (81.1)	142 (72.8)	**0.021^a^**
**Levofloxacin, n (%)**	290	229 (58.4)	61 (31.3)	**<0.001^a^**
**Vitamin C, n (%)**	554	379 (96.7)	175 (89.7)	**0.001^a^**
**Vitamin D, n (%)**	574	386 (98.5)	188 (96.4)	0.137
**Neurobion, n (%)**	434	321 (81.9)	113 (57.9)	**<0.001^a^**
**Zinc, n (%)**	530	373 (95.2)	157 (80.5)	**<0.001^a^**
**Dexamethasone, n (%)**	308	270 (68.9)	38 (19.5)	**<0.001^a^**
**Tocilizumab, n (%)**	14	14 (3.6)	0 (0.0)	**0.007^a^**
**IVIG, n (%)**	5	5 (1.3)	0 (0.0)	0.176

^a^ *p*-value <0.05 is considered statistically significant.

ALT, alanine aminotransaminase; AST, aspartate aminotransferase; BMI, body mass index; BUN, blood urea nitrogen; COVID-19, coronavirus disease 2019; cQT, corrected QT; eGFR, estimated glomerular filtration rate; IQR, interquartile range; IVIG, intravenous Immunoglobulin; MAP, mean arterial pressure; N, total sample.

The bold values indicate the *p*-value less than 0.05 is considered significant.

**FIGURE 2:**
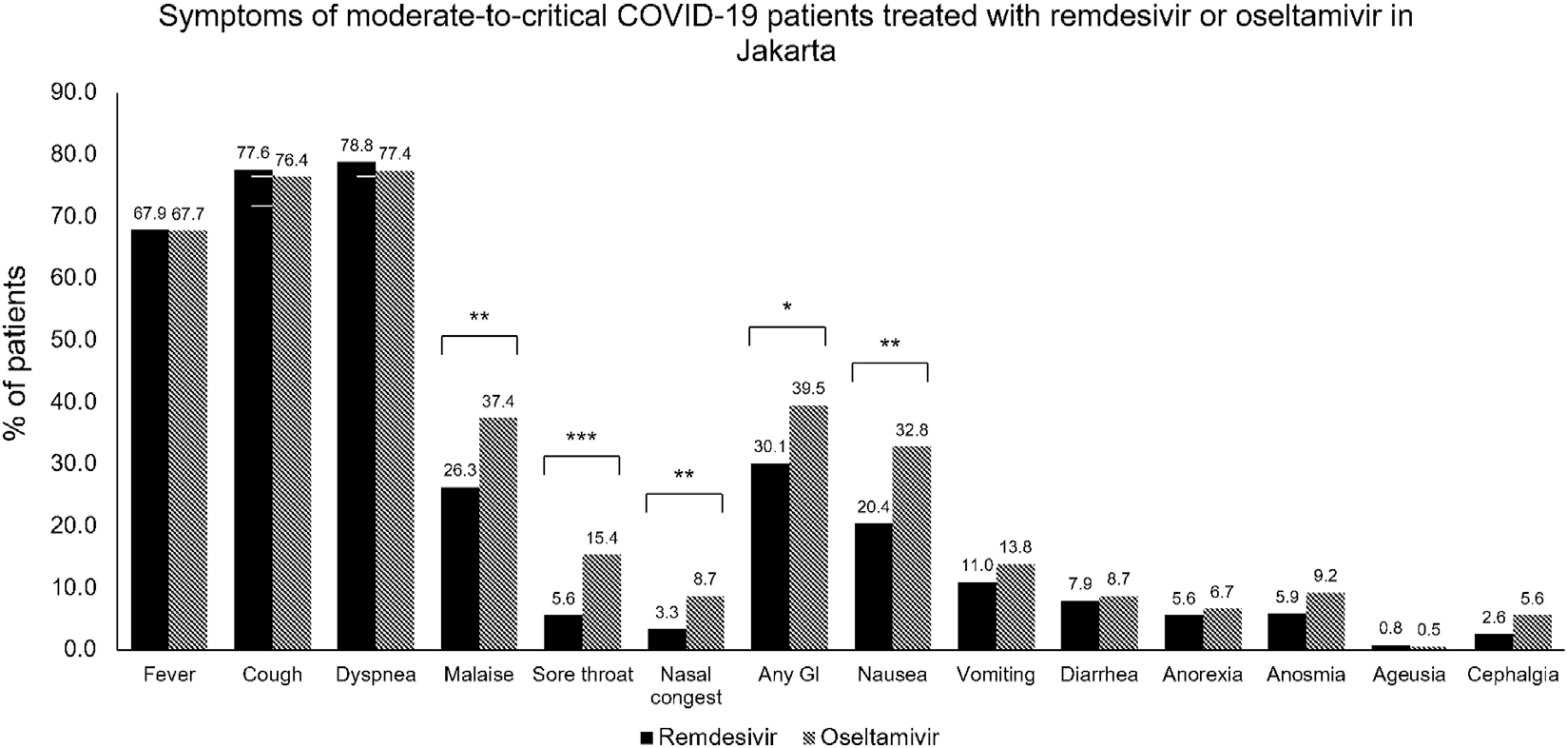
Symptoms of moderate-to-critical COVID-19 patients treated with remdesivir or oseltamivir in Jakarta. COVID-19, COronaVIrus Disease of 2019; GI, gastrointestinal symptoms. *p < 0.05; **p < 0.01; ***p < 0.001.

As seen in Table 1, most of the patients presented with moderate severity (52.6%). The remdesivir group had a higher percentage of severe to critical patients: 221 (56.4%) versus 50 (25.6%) in the control group. Patients with mild severity were not enrolled in the control group study to ensure the validity of the study. Dyspnea was the most common sign at admission, observed in 76.8% of the remdesivir group and 77.4% of the control group. More than half had sign or symptom of cough (77.6% vs 76.4%) and had sign of fever (67.9% vs 67.7%); followed by malaise, gastrointestinal symptoms, nausea, sore throat, vomiting, diarrhea, anosmia, anorexia, cephalgia, and ageusia as seen in Figure 2.

**FIGURE 3:**
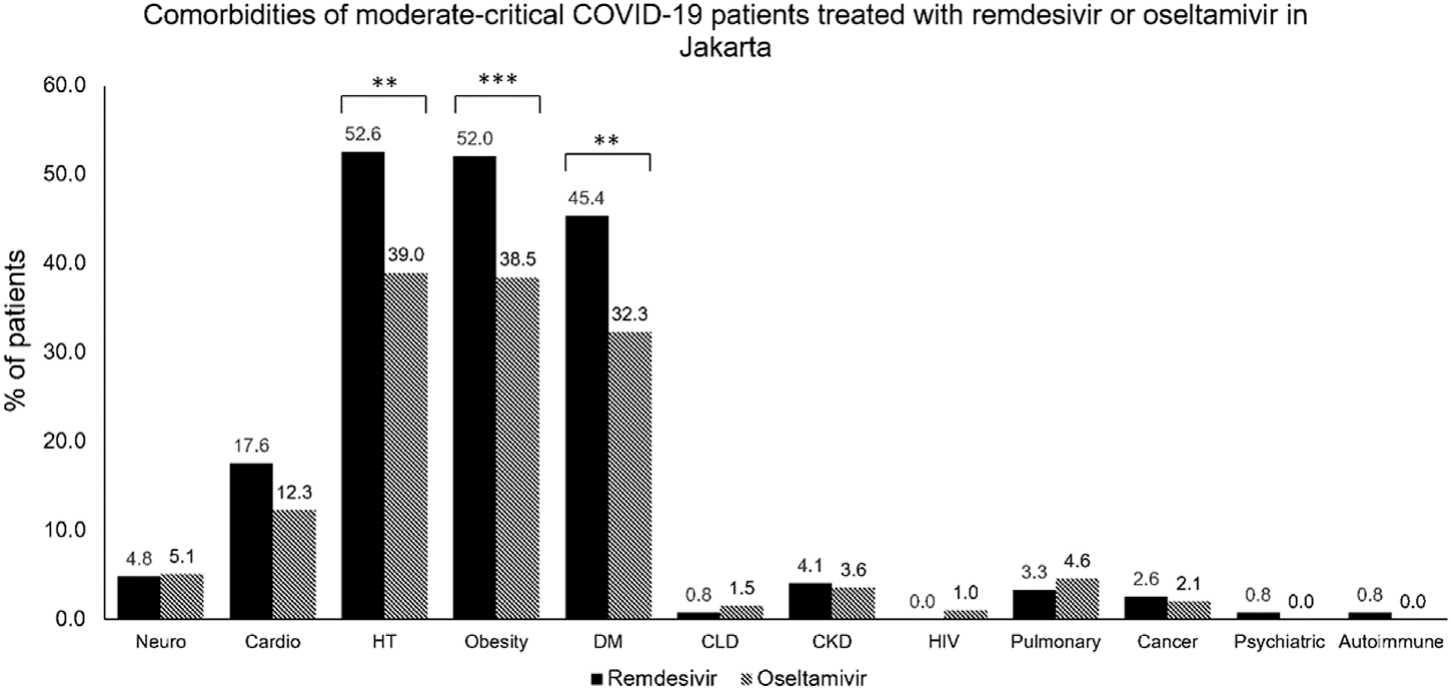
Comorbidities of moderate-to-critical COVID-19 patients treated with remdesivir or oseltamivir in Jakarta. CKD, chronic kidney disease; CLD, chronic lung disease; COVID-19, COronaVIrus Disease of 2019; DM, diabetes mellitus; HIV, human immunodeficiency virus; HT, hypertension. *p < 0.05; **p < 0.01; ***p < 0.001.

As seen in Table 1, this study also evaluated liver function through SGOT and SGPT, renal function through blood urea nitrogen, creatinine, and eGFR, and the corrected QT interval. Some of the study sites did not routinely perform all laboratory examinations and ECGs; hence, there were missing values. This study also assessed the comorbidities of the subjects enrolled, as seen in Table 1 and Figure 3. Hypertension was the most frequently reported underlying disease in the remdesivir group and control group, at 52.6% and 39.0%, respectively, followed by obesity (52% vs 38.5%), type-2 diabetes mellitus (45.4% vs 32.3%), cardiovascular disease (17.6% vs 12.3%), neurological disease (4.8% vs 5.1%), and chronic kidney disease (4.1% vs 3.6%). In terms of medication used, about more than half of all patients (64.4%) received corticosteroid therapy, and the proportion of patients receiving therapy was different between the two groups (remdesivir group: 81.9% vs Control Group: 29.2%). The remdesivir group received tocilizumab (10.2%) or intravenous immunoglobulin (2.8%), while none of the control group did (*p* < 0.001 and = 0.019).

This study shows no cases of hypersensitivity or anaphylaxis. At least one AE was recorded for most patients (69.7%), as seen in Table 2. The most common grade 1–2 AEs were anemia (18.6%), alkalosis (10.7%), and hypokalemia (8.2%). At baseline, 54 (22.2%) and 22 (6.4%) patients in the treatment and control groups, respectively, were in the ICU. There was only one case of urticaria in the control group. There were two cases of headache recorded in the remdesivir group (grades 1 and 2). QT prolongation cases were unable to be assessed since there were only three ECGs before and after treatment and there was no QT prolongation found. A single case of hallucination in the remdesivir group (grade 2) was recorded.

**TABLE 2:** Adverse event, mortality, and ventilator use.

Adverse event	N	Remdesivir	Oseltamivir	*p*-value	OR	Lower CI	Upper CI
**Physical (yes/no)**							
**Nausea, n (%)**	7	6 (2.0)	1 (0.9)	0.682	2.13	1.01	4.50
**Diarrhea, n (%)**	4	2 (0.7)	2 (1.8)	0.282	0.36	0.13	0.95
**Hypotension, n (%)**	31	25 (8.1)	6 (5.5)	0.367	1.48	1.03	2.13
**Hypertension, n (%)**	74	52 (16.9)	22 (20.2)	0.447	0.84	0.65	1.08
**Delirium, n (%)**	2	1 (0.3)	1 (0.9)	0.456	0.36	0.09	1.42
**Unconsciousness, n (%)**	2	1 (0.4)	1 (1.0)	0.491	0.40	0.10	1.61
**Laboratory (yes/no)**							
**Elevated ALT, n (%)**	15	9 (6.0)	6 (14.6)	0.095	0.41	0.24	0.69
**Elevated AST, n (%)**	12	8 (5.3)	4 (9.8)	0.286	0.54	0.30	0.97
**Decrease eGFR, n (%)**	30	27 (19.7)	3 (9.1)	0.151	2.17	1.46	3.22
**Lymphopenia, n (%)**	11	7 (2.5)	4 (4.8)	0.290	0.53	0.29	0.97
**Neutropenia, n (%)**	1	1 (0.4)	0 (0.0)	1.000	-	-	-
							
**Mortality, n (%)**				**<0.001^a^**	3.01	1.94	4.68
**Death**	141	121 (30.9)	20 (10.3)				
**Alive**	446	271 (69.1)	175 (89.7)				
							
**LOS discharge, n (%)**				0.127	1.42	0.90	2.24
**≤14 days**	337	198 (73.1)	139 (79.4)				
**>14 days**	109	73 (26.9)	36 (20.6)				
							
**LOS mild-moderate, n (%)**				0.139	1.62	0.85	3.09
**≤14 days**	235	115 (80.4)	120 (87.0)				
**>14 days**	46	28 (19.6)	18 (13.0)				
							
**LOS severe-critical, n (%)**				0.137	0.57	0.27	1.19
**≤14 days**	102	83 (64.8)	19 (51.4)				
**>14 days**	63	45 (35.2)	18 (48.6)				
							
**LOS death, n (%)**				0.865	1.09	0.40	2.95
**≤14 days**	94	81 (57.4)	13 (9.2)				
**>14 days**	47	40 (28.4)	7 5)				
							
**Time to death, median (IQR)**	587	11.3 (10.6–12.0)	10.9 (10.07–12.9)	0.645	-	-	-
							
**O2 Supplementation, n (%)**				**0.009^a^**	2.69	1.27	5.65
**Ventilator**	356	42 (7.7)	314 (57.5)				
**No Ventilator**	190	9 (1.6)	181 (33.2)				

^a^ *p*-value <0.05 is considered statistically significant.

ALT, alanine aminotransaminase; AST, aspartate aminotransferase; CI, confidence interval; eGFR, estimated glomerular filtration rate; LOS, length of stay; N, total sample; OR, odd ratio.

The bold values indicate the *p*-value less than 0.05 is considered significant.

Based on logistic regression in Table 2, mild, severe, and critical severity at admission were significantly associated with mortality (p 0.001). Remdesivir alone is not a factor leading to death, as severity is associated significantly with death, and remdesivir is the drug of choice for severe and critical cases of COVID-19. As shown in Table 2, this study also recorded the length of stay (LOS) until alive discharge (in days) for all eligible patients. The percentage of prolonged LOS (defined as LOS more than 14 days) in the remdesivir group was less than the control group, even though there was no significance found in this study. Among COVID-19 patients who were admitted with mild-to-moderate disease at baseline, patients in the remdesivir group needed to be hospitalized longer and discharged alive than the control group (19.6% vs 13%), but there were no significant associations found. Among severe and critical COVID-19 patients, more patients in the remdesivir group could be discharged alive in 14 days or less, with the percentage of prolonged stays (more than 14 days) being higher in the remdesivir group than in the control group (35.2% vs 48.6%). Even though there was no significant association found (*p* = 0.137), the confidence interval of the odds ratio is narrow (OR 0.572 (0.273–1.199), suggesting remdesivir might be beneficial to the patient.

The length of hospitalization until death was recorded in Table 2. Our study shows that the remdesivir group did not show any significant association with the LOS until death (*p* = 0.865). Patients in the remdesivir group reached mortality at an average of 11.3 days (95% CI, 10.6–12) and those in the control group reached mortality at an average of 10.9 days (95% CI, 10.07–12.9). There was no significant difference found. This result might be due to the severity of COVID-19 when the patients received remdesivir, most of whom were already in severe or critical cases. In our study, there was a significant association between remdesivir and the need for mechanical ventilation among COVID-19 patients (*p* = 0.009), as shown in Table 2. This result might be since patients who received remdesivir were already in a severe or critical state, so mechanical ventilation was needed. Because some hospitals in our study had a lower capacity for ICUs, the need for mechanical ventilation varies by hospital.

## Discussion

In this study, patients receiving remdesivir within 72 h of admission exhibited different demographic and clinical characteristics than the control group. These differences may have implications for the safety and efficacy of remdesivir in patients with moderate-to-severe COVID-19 infection. Compared to the control group, the remdesivir group had a greater median age. This finding is consistent with previous research indicating a higher risk of severe disease, increased mortality, and prolonged hospitalization in older COVID-19 patients (Li et al., 2020; Zheng et al., 2020). Age-related factors like immunosenescence and the presence of multiple comorbidities may contribute to the poorer outcomes observed in this population (Tizazu et al., 2022). Understanding the impact of age on the efficacy of remdesivir treatment is crucial for customizing therapeutic strategies and enhancing patient outcomes. In the remdesivir group, the proportion of male patients was also higher, according to the study. This is consistent with the existing literature, which has identified masculine gender as a risk factor for severe COVID-19 infections and higher mortality rates (Lipsky and Hung, 2020). Biological differences between men and women, such as variations in immune response and the impact of sex hormones, may explain these disparities in disease severity and clinical outcomes (Lipsky and Hung, 2020; Raza et al., 2021). In light of this, considering gender differences in the context of remdesivir treatment may provide valuable insight into the drug’s potential benefits and limitations in various patient populations. Compared to the control group, the remdesivir group had a greater proportion of patients with severe or critical COVID-19 disease. This is an important consideration when assessing the efficacy of remdesivir, as the drug’s impact on patient outcomes may vary based on the disease’s stage. Previous research indicates that early administration of remdesivir may be more effective at reducing viral load and enhancing clinical outcomes (Hussain Alsayed et al., 2021; Biancofiore et al., 2022; Gottlieb et al., 2022). Consequently, the schedule of remdesivir treatment concerning disease severity may be a crucial factor in determining its efficacy (Hussain Alsayed et al., 2021). In addition, the remdesivir group had a higher prevalence of comorbidities such as obesity, diabetes, and at least one cardiac or vascular disease than the control group. These comorbidities are known to increase the risk of COVID-19 severity and negative outcomes (Russell et al., 2023). The presence of these comorbidities may affect the pharmacokinetics and pharmacodynamics of remdesivir, potentially affecting the drug’s safety and effectiveness (Tumminia et al., 2022). In addition, the management of these comorbidities may necessitate the use of concomitant medications that may interact with remdesivir and influence patient outcomes (Shini Rubina et al., 2022; Tumminia et al., 2022).

In this investigation, there were no reports of hypersensitivity or anaphylaxis, indicating that remdesivir was generally well tolerated by the study population. However, the majority of patients (69.7%) experienced at least one adverse event. Anemia, alkalosis, and hypokalemia were the most frequently reported AEs in grades 1–2. During treatment with remdesivir, it is crucial to monitor and manage these adverse events to prevent potential complications and guarantee patient safety (Wu et al., 2022). Anemia is a known adverse effect of remdesivir, as the drug has been reported to temporarily reduce hemoglobin levels (Gupta et al., 2020; Nabati and Parsaee, 2022). Mild to moderate anemia may not have a significant impact on patient outcomes; however, severe anemia may exacerbate underlying conditions or contribute to the onset of new complications (Bergamaschi et al., 2021). Clinicians should be vigilant in monitoring hemoglobin levels during treatment with remdesivir and should consider appropriate interventions as needed. Alkalosis, another reported AE in this study, may be caused by a variety of factors, including respiratory or metabolic factors. Although generally benign, severe or persistent alkalosis can result in complications such as arrhythmias, convulsions, and decreased tissue oxygenation (Mirrakhimov et al., 2017). Identifying and treating the underlying causes of alkalosis during treatment with remdesivir is crucial for minimizing the risk of adverse effects. Patients receiving remdesivir also demonstrated hypokalemia. This electrolyte imbalance may be caused by several factors, such as gastrointestinal losses, renal losses, or transcellular shifts (Alfano et al., 2021; Pourfridoni et al., 2021). Hypokalemia can have severe clinical consequences, including muscle weakness, paralysis, and life-threatening cardiac arrhythmias (Alfano et al., 2021). Monitoring and adjusting potassium levels during treatment with remdesivir is essential for preventing significant complications. The study reported one case of urticaria in the control group, two cases of pain in the remdesivir group, and one case of hallucination in the remdesivir group. When evaluating the overall safety profile of remdesivir, these adverse events should be considered, despite their rarity. In addition, QT prolongation could not be adequately assessed due to the limited ECG data available. Future studies should further investigate the potential impact of remdesivir on the QT interval and associated hazards.

In this study, logistic regression analysis demonstrated that disease severity (moderate, severe, and critical) at admission was significantly associated with mortality (p 0.001). This finding is consistent with previous research demonstrating that the severity of COVID-19 is a crucial predictor of patient outcomes, such as mortality (Esfahanian et al., 2021; Salaffi et al., 2021; Kowsar et al., 2023). However, the analysis did not reveal a significant association between treatment with remdesivir alone and mortality. This result may be attributable to the fact that remdesivir was predominantly administered to patients with severe or critical COVID-19, a population with an inherently increased mortality risk. Although a statistically significant difference in mortality was observed between the remdesivir group and the control group, it is essential to consider the impact of confounding variables, such as patient demographics, comorbidities, and concomitant medications, on the study outcomes. These variables may have contributed to the observed mortality rates and may have obscured the true effect of remdesivir on patient survival. Further research is required to compensate for these confounding variables and better understand the role of remdesivir in reducing mortality among COVID-19 patients. The timing of remdesivir administration may also play an important role in determining its efficacy in reducing mortality. Several studies suggest that early treatment with remdesivir may result in enhanced clinical outcomes and decreased mortality rates (Hussain Alsayed et al., 2021; Chokkalingam et al., 2022). Therefore, future research should examine the optimal timing of remdesivir initiation concerning disease severity to maximize its potential benefits.

In this study, the percentage of prolonged LOS was lower in the remdesivir group than in the control group, although no statistically significant difference was observed. Among COVID-19 patients admitted with mild to moderate disease at baseline, more patients in the remdesivir group required extended hospitalization and were discharged alive than in the control group (19.6% vs 13%). However, no significant association was observed between remdesivir treatment and LOS in this subgroup of patients. Intriguingly, among patients with severe and critical COVID-19, more patients in the remdesivir group were discharged alive within 14 days or less than those in the control group. 35.2% of patients in the remdesivir group remained hospitalized for longer than 14 days, compared to 48.2% in the control group. Although no significant association was discovered (*p* = 0.137), the narrow confidence interval of the odds ratio [OR 0.572 (0.273–1.199)] suggests that remdesivir may be advantageous for these patients. These results suggest that the effect of remdesivir on LOS may be more pronounced in certain patient subgroups, specifically those with severe or critical COVID-19. In addition to patient demographics, comorbidities, disease severity, and the schedule of remdesivir initiation, more research is required to elucidate the factors contributing to these observed differences in LOS.

There was a significant association between remdesivir treatment and the need for mechanical ventilation among COVID-19 patients in this study (*p* = 0.009). This result may be attributable to the fact that patients who received remdesivir were already in a severe or catastrophic condition that required mechanical ventilation. It is essential to note that some hospitals included in the study had limited intensive care unit (ICU) capacity, which may have influenced the use of ventilators at various facilities. Tethodshe association between remdesivir treatment and ventilator use raises several concerns about the drug’s ability to reduce the need for mechanical ventilation in certain patient subgroups. In addition, when evaluating the efficacy of remdesivir, the influence of concomitant medications and supportive care measures on the use of a ventilator must be considered. For example, the use of corticosteroids, tocilizumab, or intravenous immunoglobulins, which have been shown to influence patient outcomes in COVID-19, may also impact the need for mechanical ventilation. The results highlight the significance of identifying patient subgroups who may derive the greatest benefit from remdesivir treatment in terms of reduced ventilator use. Understanding the characteristics of these subgroups can assist in optimizing therapeutic strategies and improving patient outcomes.

When interpreting the findings, however, several limitations must be considered. The observational nature of the study design may introduce potential biases, such as confounding variables and selection bias. Although the researchers attempted to account for confounding variables using statistical analyses, unmeasured or unknown confounders may still have influenced the study’s results. Randomized controlled trials would provide stronger evidence, but observational designs are still useful for assessing efficacy and safety in the real world (Sharma et al., 2019). Our research methodology is predominately based on a retrospective analysis of patient data, a framework that inherently entails certain limitations. This approach, while practical and sometimes necessary, can introduce potential biases and confounding factors. In addition, we recognize that the lack of a placebo-controlled randomized trial hinders our ability to establish a direct causal relationship between Remdesivir and the observed patient outcomes. These limitations highlight the need for additional research, notably prospective, randomized, and controlled trials, to confirm our findings and provide more conclusive proof of Remdesivir’s efficacy in treating COVID-19 patients. When we compare our results with those of other studies, it is important to consider these limitations, as well as differences in patient cohorts, treatment protocols, and the variable course of COVID-19 itself. Our study fits within the broader research context, and contributes to the ongoing global effort to understand and effectively combat this virus.

Our study was primarily focused on short-term outcomes, given the relative recency of COVID-19 as a public health concern. The potential long-term effects of Remdesivir administration are indeed an area that needs further investigation. As our understanding of COVID-19 continues to evolve, so too must our understanding of its treatment options. The long-term impacts of both the infection and its treatments are critical areas of future research. We hope that our study can act as a stepping stone for such further investigations into the long-term safety and effectiveness of Remdesivir. m.

Despite these limitations, the study contributes to the expanding body of evidence on the safety and efficacy of remdesivir in the treatment of moderate-to-critical COVID-19 patients. Future research should address these limitations by employing more robust study designs, including a broader spectrum of patient populations, and collecting comprehensive data to further elucidate the role of remdesivir in COVID-19 treatment and improve patient outcomes.

## Conclusion

The study concludes that remdesivir is safe and effective in the treatment of moderate-to-critical COVID-19 in a real-world setting in Indonesia.

## Data Availability

The original contributions presented in the study are included in the article/Supplementary Material, further inquiries can be directed to the corresponding author.
