# Supplementary material for: Evaluation of safety and effectiveness of remdesivir in treating COVID-19 patients after emergency use authorization study
Source: Front Pharmacol. 2023 Jun 30;14:1205238. doi: 10.3389/fphar.2023.1205238 (PMC10347402; doi:10.3389/fphar.2023.1205238)
Supplement: Supplementary file 1 [file DataSheet1.docx]

**Supplementary table 1.** Factors associated with adverse events among adult hospitalized COVID-19 patients in Jakarta

| Characteristic | cRR (95% CI) | p-value | aRR (95% CI) | p-value |
| --- | --- | --- | --- | --- |
| Early remdesivir treatment | 1.09 (0.98–1.21) | 0.113 | 1.00 (0.91–1.11) | 0.956 |
| Age >65 years | 1.19 (1.07–1.33) | 0.004 | 1.10 (0.99–1.22) | 0.088 |
| Female sex | 0.91 (0.82–1.02) | 0.106 | 0.98 (0.88–1.10) | 0.777 |
| Diabetes mellitus | 1.22 (1.10–1.36) | <0.001 | 1.11 (1.00–1.23) | 0.047 |
| Any cardiac/vascular disease | 1.17 (1.05–1.30) | 0.005 | 1.10 (0.99–1.22) | 0.077 |
| Obesity | 1.07 (0.96–1.19) | 0.231 | 1.03 (0.93–1.15) | 0.522 |
| Hypotension at baseline | 1.46 (1.38–1.54) | 0.003 | 1.29 (1.15–1.44) | <0.001 |
| Baseline RR ≥30 breaths/minute | 1.24 (1.10–1.39) | 0.004 | 1.07 (0.95–1.20) | 0.258 |
| Severe/critical disease at baseline | 1.40 (1.26–1.56) | <0.001 | 1.27 (1.13–1.41) | <0.001 |
| Thrombocytopenia at baseline | 1.32 (1.16–1.50) | 0.010 | 1.15 (1.00–1.32) | 0.048 |
| Elevated baseline ALT | 1.30 (1.16–1.46) | <0.001 | 1.18 (1.06–1.32) | 0.003 |
| Elevated baseline BUN | 1.35 (1.23–1.48) | <0.001 | 1.17 (1.06–1.29) | 0.001 |

Bivariate association was assessed with Chi-squared test. Effect sizes were given in crude and adjusted risk ratios (cRR and aRR). ALT, alanine transaminase; BUN, blood urea nitrogen. Obesity was defined as body mass index >25 kg/m^2^. Hypotension was defined as systolic blood pressure <90 mmHg or diastolic blood pressure <60 mmHg. Thrombocytopenia was defined as thrombocyte count <150,000/µL. Upper limit of normal for aspartate transaminase and blood urea nitrogen were 34 unit/L and 20.6 mg/dL, respectively.

**Supplementary table 2.** Factors associated with cardiovascular adverse events among adult hospitalized COVID-19 patients in Jakarta

| Characteristic | cRR (95% CI) | p-value | aRR (95% CI) | p-value |
| --- | --- | --- | --- | --- |
| Early remdesivir treatment | 0.82 (0.61–1.10) | 0.172 | 0.74 (0.55–0.99) | 0.044 |
| Age >65 years | 1.44 (1.07–1.94) | 0.020 | 1.17 (0.86–1.58) | 0.309 |
| Female sex | 1.10 (0.82–1.46) | 0.532 | 1.16 (0.87–1.54) | 0.317 |
| Diabetes mellitus | 1.38 (1.04–1.82) | 0.026 | 1.21 (0.90–1.62) | 0.201 |
| Any cardiac/vascular disease | 1.31 (0.98–1.75) | 0.064 | 1.21 (0.90–1.63) | 0.198 |
| Obesity | 0.76 (0.57–1.01) | 0.059 | 0.75 (0.56–1.00) | 0.047 |
| Hypotension at baseline | 2.33 (1.52–3.55) | 0.003^a^ | 1.78 (1.15–2.76) | 0.009 |
| Baseline RR ≥30 breaths/minute | 1.40 (0.99–2.00) | 0.069 | 1.20 (0.84–1.71) | 0.325 |
| Severe/critical disease at baseline | 1.69 (1.26–2.25) | <0.001 | 1.50 (1.10–2.06) | 0.011 |
| Thrombocytopenia at baseline | 1.62 (1.01–2.58) | 0.063 | 1.28 (0.82–2.01) | 0.279 |
| Elevated baseline ALT | 1.41 (1.05–1.90) | 0.019 | 1.31 (0.96–1.78) | 0.089 |
| Elevated baseline BUN | 1.58 (1.18–2.12) | 0.003 | 1.27 (0.94–1.73) | 0.121 |

Bivariate association was assessed with Chi-squared test unless otherwise specified. Effect sizes were given in crude and adjusted risk ratios (cRR and aRR). ALT, alanine transaminase; BUN, blood urea nitrogen. Obesity was defined as body mass index >25 kg/m^2^. Hypotension was defined as systolic blood pressure <90 mmHg or diastolic blood pressure <60 mmHg. Thrombocytopenia was defined as thrombocyte count <150,000/µL. Upper limit of normal for aspartate transaminase and blood urea nitrogen were 34 unit/L and 20.6 mg/dL, respectively.

**Supplementary table 3**. Factors associated with respiratory adverse events among adult hospitalized COVID-19 patients in Jakarta

| Characteristic | cRR (95% CI) | p-value | aRR (95% CI) | p-value |
| --- | --- | --- | --- | --- |
| Early remdesivir treatment | 0.69 (0.49–0.97) | 0.029 | 0.56 (0.40–0.78) | 0.001 |
| Age >65 years | 1.35 (0.96–1.89) | 0.092 | 1.12 (0.81–1.55) | 0.479 |
| Female sex | 0.69 (0.49–0.97) | 0.027 | 0.78 (0.57–1.08) | 0.133 |
| Diabetes mellitus | 1.50 (1.10–2.05) | 0.010 | 1.22 (0.91–1.64) | 0.187 |
| Any cardiac/vascular disease | 1.33 (0.97–1.83) | 0.075 | 1.34 (1.00–1.80) | 0.049 |
| Obesity | 1.07 (0.78–1.46) | 0.683 | - | - |
| Hypotension at baseline | 1.56 (0.76–3.20) | 0.207^a^ | 1.20 (0.56–2.57) | 0.640 |
| Baseline RR ≥30 breaths/minute | 1.84 (1.27–2.66) | 0.003 | 1.42 (1.01–2.02) | 0.047 |
| Severe/critical disease at baseline | 3.05 (2.17–4.29) | <0.001 | 2.69 (1.89–3.82) | <0.001 |
| Thrombocytopenia at baseline | 1.86 (1.13–3.07) | 0.029 | 1.44 (0.89–2.32) | 0.140 |
| Elevated baseline ALT | 1.71 (1.23–2.36) | <0.001 | 1.35 (0.98–1.86) | 0.070 |
| Elevated baseline BUN | 2.00 (1.46–2.73) | <0.001 | 1.38 (1.00–1.90) | 0.049 |

Bivariate association was assessed with Chi-squared test unless otherwise specified. Effect sizes were given in crude and adjusted risk ratios (cRR and aRR). ALT, alanine transaminase; BUN, blood urea nitrogen. Obesity was defined as body mass index >25 kg/m^2^. Hypotension was defined as systolic blood pressure <90 mmHg or diastolic blood pressure <60 mmHg. Thrombocytopenia was defined as thrombocyte count <150,000/µL. Upper limit of normal for aspartate transaminase and blood urea nitrogen were 34 unit/L and 20.6 mg/dL, respectively.

**Supplementary table 4.** Factors associated with grade ≥3 adverse events among adult hospitalized COVID-19 patients in Jakarta

| Characteristic | cRR (95% CI) | p-value | aRR (95% CI) | p-value |
| --- | --- | --- | --- | --- |
| Early remdesivir treatment | 0.94 (0.77–1.15) | 0.554 | 0.78 (0.64–0.94) | 0.008 |
| Age >65 years | 1.50 (1.22–1.83) | <0.001 | 1.23 (1.03–1.48) | 0.026 |
| Female sex | 0.77 (0.62–0.95) | 0.013 | 0.87 (0.71–1.06) | 0.179 |
| Diabetes mellitus | 1.48 (1.22–1.81) | <0.001 | 1.20 (0.99–1.45) | 0.059 |
| Any cardiac/vascular disease | 1.46 (1.18–1.79) | <0.001 | 1.33 (1.10–1.61) | 0.004 |
| Obesity | 1.02 (0.84–1.25) | 0.824 | - | - |
| Hypotension at baseline | 1.95 (1.48–2.56) | 0.001 | 1.49 (1.12–1.99) | 0.007 |
| Baseline RR ≥30 breaths/minute | 1.62 (1.31–2.01) | <0.001 | 1.17 (0.96–1.43) | 0.110 |
| Severe/critical disease at baseline | 2.49 (1.99–3.11) | <0.001 | 2.11 (1.68–2.66) | <0.001 |
| Thrombocytopenia at baseline | 1.68 (1.27–2.23) | 0.004 | 1.29 (1.00–1.66) | 0.046 |
| Elevated baseline ALT | 1.75 (1.40–2.18) | <0.001 | 1.36 (1.10–1.69) | 0.005 |
| Elevated baseline BUN | 1.89 (1.57–2.28) | <0.001 | 1.35 (1.12–1.63) | 0.002 |

Bivariate association was assessed with Chi-squared test unless otherwise specified. Effect sizes were given in crude and adjusted risk ratios (cRR and aRR). ALT, alanine transaminase; BUN, blood urea nitrogen. Obesity was defined as body mass index >25 kg/m^2^. Hypotension was defined as systolic blood pressure <90 mmHg or diastolic blood pressure <60 mmHg. Thrombocytopenia was defined as thrombocyte count <150,000/µL. Upper limit of normal for aspartate transaminase and blood urea nitrogen were 34 unit/L and 20.6 mg/dL, respectively.

**Supplementary table 5.** Factors associated with hematologic adverse events among adult hospitalized COVID-19 patients in Jakarta

| Characteristic | cRR (95% CI) | p-value | aRR (95% CI) | p-value |
| --- | --- | --- | --- | --- |
| Early remdesivir treatment | 1.03 (0.83–1.28) | 0.773 | 0.89 (0.71–1.10) | 0.271 |
| Age >65 years | 1.29 (1.03–1.63) | 0.036 | 1.07 (0.85–1.35) | 0.577 |
| Female sex | 0.76 (0.60–0.96) | 0.018 | 0.89 (0.71–1.12) | 0.330 |
| Diabetes mellitus | 1.38 (1.12–1.71) | 0.003 | 1.16 (0.94–1.43) | 0.168 |
| Any cardiac/vascular disease | 1.47 (1.18–1.85) | <0.001 | 1.38 (1.10–1.72) | 0.005 |
| Obesity | 1.13 (0.91–1.41) | 0.262 | - | - |
| Hypotension at baseline | 1.12 (0.65–1.93) | 0.701 | - | - |
| Baseline RR ≥30 breaths/minute | 1.50 (1.17–1.93) | 0.003 | 1.19 (0.94–1.52) | 0.147 |
| Severe/critical disease at baseline | 1.68 (1.35–2.10) | <0.001 | 1.37 (1.09–1.72) | 0.007 |
| Thrombocytopenia at baseline | 1.77 (1.31–2.40) | 0.003 | 1.41 (1.04–1.92) | 0.027 |
| Elevated baseline ALT | 1.90 (1.49–2.42) | <0.001 | 1.64 (1.29–2.10) | <0.001 |
| Elevated baseline BUN | 1.72 (1.39–2.13) | <0.001 | 1.36 (1.09–1.70) | 0.006 |

Bivariate association was assessed with Chi-squared test unless otherwise specified. Effect sizes were given in crude and adjusted risk ratios (cRR and aRR). ALT, alanine transaminase; BUN, blood urea nitrogen. Obesity was defined as body mass index >25 kg/m^2^. Hypotension was defined as systolic blood pressure <90 mmHg or diastolic blood pressure <60 mmHg. Thrombocytopenia was defined as thrombocyte count <150,000/µL. Upper limit of normal for aspartate transaminase and blood urea nitrogen were 34 unit/L and 20.6 mg/dL, respectively.

**Supplementary table 6.** Factors associated with metabolic adverse events among adult hospitalized COVID-19 patients in Jakarta

| Characteristic | cRR (95% CI) | p-value | aRR (95% CI) | p-value |
| --- | --- | --- | --- | --- |
| Early remdesivir treatment | 1.13 (0.90–1.41) | 0.293 | 0.97 (0.78–1.21) | 0.801 |
| Age >65 years | 1.30 (1.02–1.65) | 0.038 | 1.13 (0.88–1.46) | 0.325 |
| Female sex | 0.95 (0.76–1.20) | 0.684 | 1.18 (0.94–1.48) | 0.146 |
| Diabetes mellitus | 1.47 (1.18–1.83) | <0.001 | 1.22 (0.98–1.53) | 0.082 |
| Any cardiac/vascular disease | 1.38 (1.10–1.74) | 0.005 | 1.22 (0.97–1.53) | 0.094 |
| Obesity | 1.31 (1.04–1.64) | 0.018 | 1.26 (1.00–1.57) | 0.047 |
| Hypotension at baseline | 1.01 (0.55–1.86) | 0.968 | - | - |
| Baseline RR ≥30 breaths/minute | 1.73 (1.36–2.19) | <0.001 | 1.44 (1.12–1.84) | 0.004 |
| Severe/critical disease at baseline | 1.58 (1.26–1.98) | <0.001 | 1.20 (0.94–1.52) | 0.137 |
| Thrombocytopenia at baseline | 1.64 (1.17–2.30) | 0.015 | 1.28 (0.92–1.79) | 0.149 |
| Elevated baseline ALT | 1.61 (1.26–2.04) | <0.001 | 1.40 (1.10–1.78) | 0.006 |
| Elevated baseline BUN | 1.99 (1.61–2.46) | <0.001 | 1.70 (1.35–2.13) | <0.001 |

Bivariate association was assessed with Chi-squared test unless otherwise specified. Effect sizes were given in crude and adjusted risk ratios (cRR and aRR). ALT, alanine transaminase; BUN, blood urea nitrogen. Obesity was defined as body mass index >25 kg/m^2^. Hypotension was defined as systolic blood pressure <90 mmHg or diastolic blood pressure <60 mmHg. Thrombocytopenia was defined as thrombocyte count <150,000/µL. Upper limit of normal for aspartate transaminase and blood urea nitrogen were 34 unit/L and 20.6 mg/dL, respectively.

**Supplementary table 7.** Factors associated with ICU admission among adult hospitalized COVID-19 patients in Jakarta

| Characteristic | cRR (95% CI) | p-value | aRR (95% CI) | p-value |
| --- | --- | --- | --- | --- |
| Early remdesivir treatment | 0.70 (0.47–1.04) | 0.070 | 0.54 (0.37–0.81) | 0.002 |
| Age >65 years | 1.02 (0.66–1.56) | 0.938 | 0.92 (0.59–1.44) | 0.728 |
| Female sex | 0.84 (0.58–1.22) | 0.362 | 0.93 (0.64–1.34) | 0.696 |
| Diabetes mellitus | 1.57 (1.10–2.25) | 0.013 | 1.39 (0.98–1.96) | 0.062 |
| Any cardiac/vascular disease | 1.33 (0.92–1.93) | 0.122 | 1.25 (0.88–1.76) | 0.207 |
| Obesity | 1.36 (0.94–1.95) | 0.098 | 1.24 (0.86–1.79) | 0.243 |
| Hypotension at baseline | 1.76 (0.84–3.69) | 0.146 | 1.49 (0.79–2.81) | 0.214 |
| Baseline RR ≥30 breaths/minute | 2.74 (1.86–4.04) | <0.001 | 1.89 (1.30–2.75) | 0.001 |
| Severe/critical disease at baseline | 2.70 (1.86–3.92) | <0.001 | 2.15 (1.47–3.14) | <0.001 |
| Thrombocytopenia at baseline | 1.20 (0.58–2.49) | 0.625 | - | - |
| Elevated baseline ALT | 1.89 (1.29–2.78) | <0.001 | 1.58 (1.06–2.34) | 0.024 |
| Elevated baseline BUN | 1.69 (1.16–2.48) | 0.008 | 1.27 (0.89–1.83) | 0.192 |

Bivariate association was assessed with Chi-squared test unless otherwise specified. Effect sizes were given in crude and adjusted risk ratios (cRR and aRR). ALT, alanine transaminase; BUN, blood urea nitrogen. Obesity was defined as body mass index >25 kg/m^2^. Hypotension was defined as systolic blood pressure <90 mmHg or diastolic blood pressure <60 mmHg. Thrombocytopenia was defined as thrombocyte count <150,000/µL. Upper limit of normal for aspartate transaminase and blood urea nitrogen were 34 unit/L and 20.6 mg/dL, respectively.
